# Approaching the Gap: Optimizing Tricuspid Valve Coaptation During Transcatheter Tricuspid Repair∗

**DOI:** 10.1016/j.jaccas.2023.101854

**Published:** 2023-04-28

**Authors:** Ana Paula Tagliari, Maurizio Taramasso, Vaikom Subramanian Mahadevan

**Affiliations:** a Cardiac Surgery Department, Hospital Mãe de Deus, Porto Alegre, Brazil; b Cardiac Surgery Department, Hospital São Lucas da Pontifícia Universidade Católica do Rio Grande do Sul, Porto Alegre, Brazil; c HerzZentrum Hirslanden Zurich Clinic of Cardiac Surgery, Zurich, Switzerland; d Division of Cardiology, University of Massachusetts Chan School of Medicine, Worcester, Massachusetts, USA

**Keywords:** 3-dimensional imaging, echocardiography, imaging, tricuspid valve, valve repair

**Figure:**
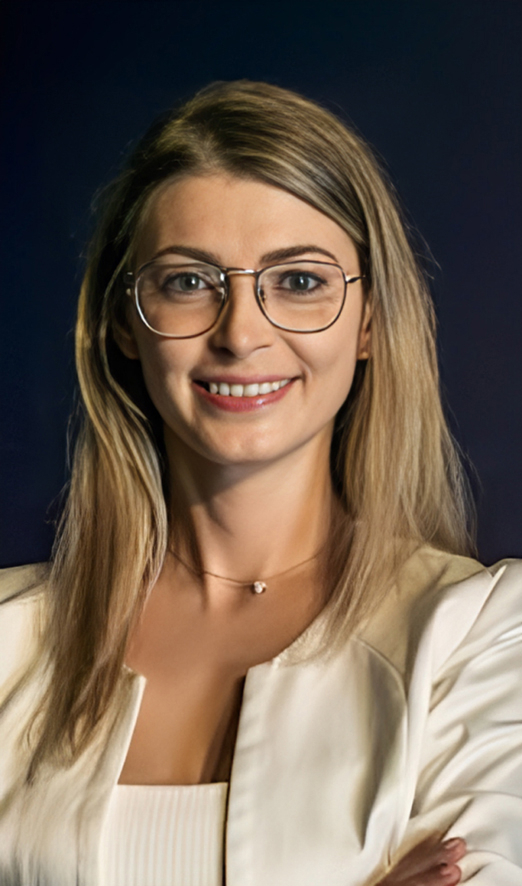

Following the widespread use of catheter-based solutions to treat aortic valve stenosis and mitral valve regurgitation, the tricuspid valve has become the focus of percutaneous techniques. However, despite the growing number of patients with severe tricuspid regurgitation (TR) who have been approached with transcatheter therapies, there are still some unmet clinical needs that limit its widespread use.

The following are some of these unmet needs: lack of strong guideline-based recommendations for TR intervention, lack of standardized patient selection criteria, challenges in imaging acquisition and the need for a multiparametric approach to systematically assess the tricuspid valve (pre-, intra-, and postoperative assessment), challenges in determining the mechanism and severity of TR, and lack of standardized endpoints and definitions (a “TVARC” consensus).^1^

Regarding transcatheter treatment options and device selection, tricuspid transcatheter edge-to-edge repair technique (T-TEER) is mostly performed using the TriClip (Abbott Vascular) or the PASCAL system (Edwards Lifesciences). Both devices, TriClip and PASCAL, are designed to allow simultaneous or independent leaflet grasping, thus optimizing leaflet approximation. Nevertheless, despite these technological facilities, intraprocedural success is highly dependent on valve anatomic features and the patients must fulfill strict echocardiographic criteria to be considered suitable for T-TEER.

Some anatomic criteria considered favorable to T-TEER are small septolateral gap ≤7 mm, anteroseptal jet location, confined prolapse or flail, and a trileaflet valve morphology. On the other hand, unfavorable anatomy is characterized by the presence of a large septolateral coaptation gap >8.5 mm, leaflet thickening/shortening (rheumatic, carcinoid)/perforation, dense chordae with marked leaflet tethering, anteroposterior jet location, poor echocardiographic leaflet visualization, the presence of a right ventricle lead causing leaflet impingement, and an unfavorable device angle of approach.^1^

Among all these variables, the coaptation gap has been considered one of the main anatomic predictors of poor technical success.^2^ From a pathophysiological point of view, the coaptation gap is likely a result of tricuspid annular dilatation and leaflet tethering, with larger coaptation gaps being associated with more severe TR and, consequently, with an increased likelihood of residual TR. A larger coaptation gap may prevent successful clip placement or lead to clip placement not exactly in the primary target lesion, resulting in suboptimal TR reduction. Besler et al^3^ identified a small TR coaptation gap size and a central/anteroseptal TR jet location as independent predictors of procedural success in patients with symptomatic TR undergoing T-TEER. The authors identified a coaptation gap cutoff value of 7.2 mm as the best discriminator for successful T-TEER, with a success rate of <30% in patients with a gap larger than 10 mm. Furthermore, successful T-TEER was the only predictor for freedom from clinical outcomes.^3^

Although the importance of the coaptation gap as a prognostic factor has been well recognized, few studies have explored intraprocedural maneuvers to optimize leaflet grasping in order to improve technical success. In this issue of *JACC: Case Reports*, Rivas et al^2^ highlighted the relevance of the coaptation gap measured on transesophageal echocardiography and described the usefulness of some intraprocedural maneuvers to enable or facilitate T-TEER in patients with excessive coaptation gap or short leaflet length by presenting 3 illustrative cases.

In the first case, a patient with torrential ventricular TR and a septal-lateral coaptation gap of 0.7 to 1.0 cm had the gap reduced to 0.3 to 0.6 cm by applying a ventilator-assisted or intraoperative Valsalva maneuver (increasing ventilatory gas flow and squeezing the breathing circuit bag temporarily, increasing intrathoracic pressure to approximately 30 mm Hg).

In the second, a torrential mixed atrial and ventricular functional TR with an anterior-posterior coaptation gap of 4.92 cm and septal-lateral coaptation gap of 1.5 to 1.8 cm was optimized by applying a positive end-expiratory pressure (PEEP) of 20 mm Hg to reduce the venous return in combination with external pressure applied to the patient’s sternum to physically narrow the lateral-to-septal dimension. The result was a decrease in the coaptation gap to 0.5 to 0.7 cm.

In the last patient, a severe atrial functional TR (permanent pacemaker lead related) with a septal-lateral coaptation gap of 0.38 cm and a short septal leaflet was managed by applying a PEEP of 15 cmH_2_O with subsequent narrowing of the tricuspid annulus. According to the authors, in this case, PEEP was used to reduce the tricuspid annulus diameter and bring the device closer to the septal leaflet after the anterior leaflet had been grasped.

Being aware of these optimizing maneuvers to facilitate leaflet approximation is essential for operators involved in transcatheter tricuspid valve management. This is especially important considering that preoperative clinical optimization strategies, such as reducing the preload and optimizing the volume status by aggressive diuretic therapy, may not be effective or available for all patients with TR, forcing operators to overcome intraprocedural technical limitations. In addition, it is important to remember that a comprehensive preoperative right ventricle and TR assessment is usually performed in an euvolemic state, which may not be the same as that presented intraoperatively.

Ventilator-assisted Valsalva maneuver, use of high PEEP, and chest compression are strategies potentially associated with an improvement in leaflet coaptation by acutely reducing the right ventricle preload, modifying tricuspid annulus dimension and shape, and, consequently, reducing the coaptation gap.^2^^,^^4^ Another additional maneuver is tilting the intervention table (feet low), which is associated with a reduction of right ventricular preload by venous pooling in the lower extremities.^5^ The effect of standardized table tilt was evaluated in a prospective single-center study of 36 patients undergoing T-TEER. After tilting the intervention table, the coaptation gap was reduced by approximately 25% (Δ −24.4%; from 5.6 ± 2.9 mm to 4.2 ± 2.1 mm; *P* < 0.001), as well as the coaptation gap area (Δ −25.4%; from 1.0 ± 0.8 cm^2^ to 0.7 ± 0.6 cm^2^; *P* < 0.001), and the annular perimeter (Δ −4.3%; from 17.0 ± 3.1 mm to 16.3 ± 2.5 mm; *P* = 0.002).^5^ Other potential techniques, such as transiently reducing or occluding the superior vena cava or the inferior vena cava return with a balloon, among others, could be considered in some cases.

Operators should also keep in mind that, if these maneuvers are done to reduce the coaptation gap, especially preoperative diuretic therapy optimization may be important to maintain the high diuretic doses for a few weeks after the procedures aiming to keep the preload under control, reduce the risk of tricuspid annulus redilation, and the consequent risk of leaflet tension and single leaflet device attachment.

In summary, the maneuvers described by Rivas et al^2^ in this article are simple and feasible, with the potential to favorably modify the right ventricle chamber anatomy and the tricuspid annulus size, and reduce large coaptation gaps, therefore facilitating TR interventional treatment.

## Funding Support and Author Disclosures

Dr Taramasso has been a consultant or the recipient of consultancy fees from Abbott, Edwards Lifesciences, Boston Scientific, Shenqi Medical, CoreMedic, 4tech, Simulands, MTEx, Cardiovalve, and MEDIRA. Dr Tagliari has received a research grant from CAPES - Brazil (Finance Code 001); and has received a speaking fee from Biotronik. Dr Mahadevan is a consultant for Edwards Lifesciences.
